# Decreased 3D observer variation with matched CT-MRI, for target delineation in Nasopharynx cancer

**DOI:** 10.1186/1748-717X-5-21

**Published:** 2010-03-15

**Authors:** Coen RN Rasch, Roel JHM Steenbakkers, Isabelle Fitton, Joop C Duppen, Peter JCM Nowak, Frank A Pameijer, Avraham Eisbruch, Johannes HAM Kaanders, Frank Paulsen, Marcel van Herk

**Affiliations:** 1 Department of Radiation Oncology, The Netherlands Cancer Institute/Antoni van Leeuwenhoekhuis, Amsterdam, The Netherlands; 2 Current address: Department of Radiation Oncology, University Medical Center Groningen, The Netherlands; 3 Current address: Department of Radiation Protection, CHU Henri Mondor, Créteil, France; 4 Department of Radiation Oncology, Erasmus MC, Rotterdam, The Netherlands; 5 Current address: Department of Radiology, Universtiy Medical Center Utrecht, Utrecht, The Netherlands; 6 Department of Radiation Oncology, University of Michigan Ann Arbor, Michigan, USA; 7 Department of Radiation Oncology, Radboud University Nijmegen Medical Centre, Nijmegen, The Netherlands; 8 Department of Radiation Oncology, University of Tübingen, Tübingen, Germany

## Abstract

**Purpose:**

To determine the variation in target delineation of nasopharyngeal carcinoma and the impact of measures to minimize this variation.

**Materials and methods:**

For ten nasopharyngeal cancer patients, ten observers each delineated the Clinical Target Volume (CTV) and the CTV elective. After 3D analysis of the delineated volumes, a second delineation was performed. This implied improved delineation instructions, a combined delineation on CT and co-registered MRI, forced use of sagittal reconstructions, and an on-line anatomical atlas.

**Results:**

Both for the CTV and the CTV elective delineations, the 3D SD decreased from Phase 1 to Phase 2, from 4.4 to 3.3 mm for the CTV and from 5.9 to 4.9 mm for the elective. There was an increase agreement, where the observers intended to delineate the same structure, from 36 to 64 surface % (p = 0.003) for the CTV and from 17 to 59% (p = 0.004) for the elective. The largest variations were at the caudal border of the delineations but these were smaller when an observer utilized the sagittal window. Hence, the use of sagittal side windows was enforced in the second phase and resulted in a decreased standard deviation for this area from 7.7 to 3.3 mm (p = 0.001) for the CTV and 7.9 to 5.6 mm (p = 0.03) for the CTV elective.

**Discussion:**

Attempts to decrease the variation need to be tailored to the specific causes of the variation. Use of delineation instructions multimodality imaging, the use of sagittal windows and an on-line atlas result in a higher agreement on the intended target.

## Introduction

Delineation of the target is one of the main remaining error sources in conformal radiation therapy [1,2]. By the nature of the procedure, delineation errors are systematic in external beam radiotherapy. Any deviation remains the same throughout the radiation course, which results in reproducible dose differences. Earlier reports on ethmoidal and maxillary sinus and nasopharyngeal tumors demonstrated a dose dependency of both observer variation and irradiation technique. Despite improvements in the latter, the impact of delineation variation remains large with regard to impact on dose to the target and to the other organs at risk [2,3].

Several efforts have been undertaken to decrease observer variation. Two main topics can be distinguished:

1 Guidelines for delineation

2 Multimodality imaging

Early guidelines for delineation of the neck levels were published by Som et al. Shortly thereafter, Robbins, Nowak and Gregoire et al published guidelines on the same topic. Currently, more than five different guidelines for delineation of the neck have been published in the international literature [1,4-9].

In an effort to reach consensus, Gregoire et al published consensus guidelines for neck delineation on behalf of the Radiation Therapy Oncology Group (RTOG) and European Organization for Research and Treatment of Cancer (EORTC) [10]. Although validation of these guidelines is still to be performed, they more than likely improve delineation agreement of the elective neck nodes. However, guidelines for the delineation of primary tumors of head and neck cancers are scarce.

Computer Tomography (CT) based delineation is the current standard of practice for conformal radiotherapy, although other imaging modalities like Magnetic Resonance Imaging (MRI) and Positron Emission Tomography (PET) have also proven their value in several tumor sites [6,11-13]. Notably, a study comparing CT, MRI, PET and pathological specimen based Gross tumor Volume (GTV) determination for larynx carcinomas demonstrated that MRI was more accurate than CT and PET and was even closer to the pathological specimen measurements that are regarded as the "gold standard" [6]. The addition of MRI to CT decreases observer variation and leads to smaller Gross Tumor Volumes, as seen in this study and others concerning this topic [2,6,11,14]. For example, the addition of PET to lung cancer observation considerably decreased the delineation variation [13,15-17]. This was demonstrated in a multiobserver study performed by Steenbakkers et al [15], in which the addition of PET to CT-based delineation particularly decreased observer variation at the interfaces towards mediastinum and hilum and in the case of atelectasis. Furthermore, the use of sagittal or coronal reconstructions during the delineation led to more agreement [15].

18 Fludeoxyglucose-positron Emission Tomograpy (FDG-PET) Imaging for Head and Neck provides functional information on the extent of the tumor [18-21]. However, its main strength lies in the detection of involved regions or (lymph node) metastasis, with an overall sensitivity of 79% [18,22]. For precise delineation of the tumor extent itself, however, it is less suitable. This is due to poorer spatial resolution, the lack of a universal threshold uptake value, and a large uptake in the brain tissue close to the primary tumor when invasion towards bone or parapharyngeal regions is suspected (i.e., when the delineation becomes difficult) [18,23]. MRI was superior to FDG-PET for showing the extent of the primary tumor in 54 nasopharyngeal cases[24].

The addition of MRI to CT-based delineation has proven its value in the delineation of the Head and Neck region and has resulted in smaller target volumes [2,6,11,14,25,26]. Especially when posterior invasion is suspected, MRI has proven to be superior to CT based staging [26]. However, the effect on observer variation is limited [6,11].

The above-mentioned studies for the Head and Neck have defined the variation in various modalities, but have not attempted to determine measures for decreasing this variation within the study, or measure the impact of any measures taken. It is the aim of the present study to determine the extent of baseline variation, to analyze the results, and then to take measures including improved delineation guidelines, multi-modality imaging, and delineation tools targeted at the specific variations found to reduce this variation. The impact of these measures will then be assessed by re-delineation.

## Materials and methods

For ten patients with nasopharynx cancer, delineation of the clinical target volume was performed by ten observers, considered experts in the field and from multiple institutions. Stages of the patients ranged from T2 (2), T3 (3), T4 (5), N0 (3), N1 (5), N2 (2). No lymph node delineation was performed. The aim of the study was to assess the observer variation in 3D in a standardized environment in two phases.

First, each observer was given the same personal computer with monitor, installed with in-house delineation software together with the patient data. In this phase, delineation was performed on contrast enhanced CT images with delineation instructions. The non-matched MRI was digitally available to the observer. Delineation on both the CTV (= visible tumor + suspected microscopic extension) and the CTV-elective (= CTV + 1 cm margin and the entire nasopharynx) was performed. Automatic 3D expansion of the CTV, with 1 cm as a starting point for the CTV elective, was supplied to the observer. The delineations, together with data on observer-computer interaction (Big Brother [13]), were then submitted through the Internet to the Netherlands Cancer Institute. These data contained information on the delineations and all computer actions of the observer such as mouse motion, window/level, delineation corrections (i.e. moving, deleting or replacing any point of the delineation during while delineating but before submission of the delineation). After volumetric and 3D analysis (see below), a meeting was organized with the observers. Results of the first delineation phase were discussed. Improvements in delineation instructions and how to implement the CT and MRI co-registration were then generated from the meeting.

To ensure that the observers would have forgotten the exact first delineation, one year thereafter the observers received a new CD with improved delineation software, improved instructions for delineations, and a co-registered CT-MRI for delineation. Furthermore, the observers were given an on-line CT atlas with the key anatomical boundaries pointed out and the normal boundaries of the nasopharynx highlighted. The observers were forced to use the sagittal and/or coronal side windows before the start of each delineation, by designating a point in the axial plane where a reconstruction was to be generated. Again, the CTV and CTV-elective were delineated by all observers for all patients.

First, the volume of each delineation and the volume of each median volume (The volume encompassed by the median surface, see next paragraph) were calculated. Then the common volume (volume common to all individual delineations in a patient) and encompassing volume (volume encompassing all the individual delineations in a patient) were calculated. Ideally, the ratio between common and encompassing volume was 1, indicating a full agreement between observers.

**Figure 1:**
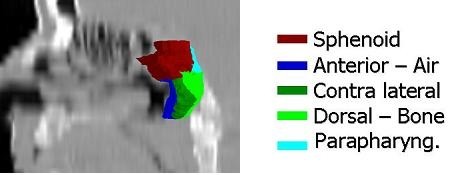
**Division of a median CTV surface in anatomical interfaces; the contralateral regions are not shown**.

Three-dimensional analysis was separately performed in both phases of the study. First, for each patient, a median surface of the delineated targets of all radiation oncologists was computed in three dimensions as described by Deurloo et al. [27]. The median surface represents 50% coverage of the delineations of all radiation oncologists, meaning that each voxel inside the median surface is designated by at least 50% of the radiation oncologists as part of the delineation. On this surface, the type of interface (i.e., tumor - air or tumor - bone) was marked manually for each case (Fig. 1). For each point describing the median surface (about 280 points/cm2), the perpendicular distance to each individual delineation was measured. When this distance was more than 2 cm or if no perpendicular distance was found, the distance to the closest point on the individual GTV was used instead. For each point describing the median surface, 10 distances (one for each observer) were measured. The variation in the 10 distances was expressed as a local standard deviation (local SD), which is a measure of local observer variation. The variation in distance to all points describing the median surface was expressed in an overall standard deviation (overall SD), a measure of overall observer variation. (Fig. 1). To combine data between patients, the quadratic sum of the standard deviations was calculated for each region separately and for all regions combined.

Complete agreement of all observers what to delineate is rare. Therefore we choose an arbitrary cutoff of 80% agreement, as described in an earlier analysis in lung cancer [15]. The median surface for each patient was manually divided into an agreement and disagreement region. The median surface was labeled as an agreement region when a corresponding anatomic structure was delineated by at least 8 of the 10 radiation oncologists (i.e., 80%); otherwise, it was labeled as a disagreement region. The regions were judged by the author and in cases of doubt the regions were judged and designated by two radiation-oncologists (CR, RS). Ideally all of the surface should be designated as agreement region as disagreement regions indicate that observer variation is determined on different opinions of the anatomical target extension rather then visibility of a structure.

## Results

A total number of 400 delineations were analyzed. The tumor volumes and standard deviations of these volumes are listed in Table 1.

**Table 1:** Volume comparison.

Target Volume	CTV el		CTV	
Delineation phase	1	2	1	2

Mean Volume (cm^3^)	103	91	25	20

Standard Deviation (cm^3^)	33	21	9	5

Mean Clinical Target Volume (CTV) and CTV elective (CTV el) comparison. First phase: delineation on CT; second phase: delineation on CT, registered MRI, forced use of sagittal reconstructions, and with improved guidelines. Standard deviations are quadratic averages from the volume standard deviation for each patient.

### CTV delineation

The mean number of corrections per CTV delineation was 51 (i.e. 9.1 corrections/cm^2^). The standard deviations of the distances from the median CTV are listed in Table 2. For the whole surface and all delineations combined, the root mean square of the standard deviation was 4.4 mm with 36% of the surface where 8/10 of the observers intended to delineate the same anatomical entity (agreement region). The largest variation was at the caudal side of the tumor i.e., perpendicular to the transverse plane of the CT scan. Only 5 percent of this caudal region could be designated an agreement region; in other words, for 95% of the caudal surface, less than 8/10 observers intended to delineate the same anatomical boundary. The second site of largest variation was towards the Sphenoid/Clivus. Here, the variation was 5 mm (1 SD), with 28% of the surface being an agreement region. For the other regions, the root mean square standard deviation of the distance from the delineations to the median volume ranged from 3.4 to 4.7 mm, with a surface percentage agreement region of 16 to 62%. The best agreement and lowest standard deviation was at the interface of tumor with air (3.4 mm, 62%).

**Table 2:** Observer variation for the various CTV to normal tissue interfaces and the two delineation phases.

Anatomical regions	Phase 1		Phase 2	
	
	SD (mm)	Agreement (%)	SD (mm)	Agreement (%)
All regions	4.4	36	3.3 (p = 0.02)	64 (p = 0.003)

Anterior - Air	3.4	62	2.7 (p = 0.01)	79 (p = 0.02)

Dorsal - Bone	3.6	49	2.7 (p = 0.005)	84 (p = 0.005)

Contra lateral	4.2	16	3.5 (p = 0.05)	66 (p = 0.004)

Pterygoid M.	4.3	35	3.1 (p = 0.02)	61 (p = 0.03)

Parapharyngeal	4.4	31	3.3 (p = 0.007)	59 (p = 0.005)

Soft Palate	4.7	37	3.0 (p = 0.005)	67 (p = 0.01)

Sphenoid	5.0	28	4.2 (p = 0.03)	48 (p = 0.01)

Caudal side	7.7	5	3.3 (p = 0.001)	56 (p < 0.001)

Overall observer variation in delineation of the CTV, quadratic mean of the SD of the distance of the delineation to the median surface as measured for each region. Agreement depicts the percentage of the surface for each region on the median surface, where the observers intended to delineate the same anatomical entity. Standard deviations are quadratic averages of patient specific standard deviations.

The second phase CTV delineation results are listed in Table 2, next to the results of the first delineation phase. The mean number of corrections per delineation was 33 (i.e., 7.3 corrections per cm^2^). The root mean square of the standard deviation of the distance between the delineations and the median surface decreased to 3.3 mm, with an agreement surface percentage of 64%. The largest root mean square SD was 4.2 mm at the sphenoid interface. In the first phase, the delineation variation between the observers using the side windows and those not using them differed from 3.7 to 5.0 mm (1SD). In the second phase, the forced use of the side windows (sagittal reconstructions) and the addition of co-registered MRI resulted in a decreased observer variation from 7.7 to 3.3 mm (1 SD) at the caudal side of the tumor. The mean delineation time decreased from 15 to 11 minutes. The mean volume of the delineations decreased from 25 to 20 cm^3 ^with an SD (including patient variation) of 9 to 5 cm^3 ^respectively. At the same time, the ratio between common and encompassing volume (i.e.: the ratio between the largest volume common to all delineations and the smallest volume encompassing all delineations) rose from 0.15 to 0.22.

The mean distance between the first and second CTV delineation of an observer and patient was 0.6 mm (SD 5.7 mm), but this was not evenly distributed. In all but one region, the second phase CTV delineations resulted in 2.6 to 0.1 mm smaller volumes. At the caudal side, the phase 2 delineation (MRI, improved delineation instructions, and the forced use of the side windows) resulted in a 1.4 mm larger mean delineation.

### CTV elective

**Figure 2:**
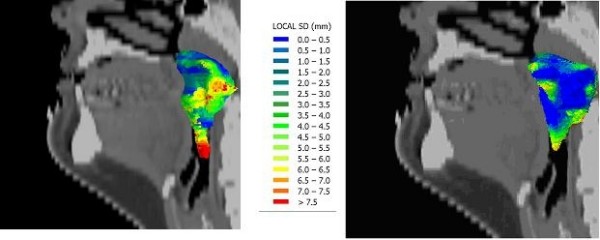
**Local delineation variation (SD) for one patient in 3D for CTV-Elective delineation Phase 1 (Left) and 2 (Right)**. Note the tail-like variation on the caudal side in the Phase 1 delineation.

The CTV elective delineation was intended to have the CTV + 1 cm margin including the nasopharynx, but corrected for delineated air or non-involved anatomical borders (i.e., towards non-involved brainstem, bone, or cerebrospinal fluid) (Fig. 2). Due to the different emphasis of the delineation, the anatomical interfaces were defined differently. As in the regions for the CTV delineations, the Pterygoid, Parapharyngeal, Sphenoid, anterior and caudal regions were designated. The dorsal side was split into regions with and regions without bone invasion. The mean number of corrections was 202 for each delineation, or 14.5/cm^2^. In part, this high number was due to the editing of the automatic expansion of the CTV in clearly non-involved regions such as cerebro-spinal fluid and air. The mean volumes are listed in Table 1.

The mean SD of the distance between the median surface and the delineations was 5.9 mm, with only 17% of the surface depicted as agreement regions (Table 3). The largest variation was again noted at the caudal border of the delineations, at 7.9 mm with an agreement surface percentage of 7%. This difference was smaller when the side windows (sagittal/coronal) views were applied. Overall, the side window usage resulted in a reduction of SD from 6.6 to 5.6 mm. The region with the least observer variation was towards the dorsal side with non-invaded bone tumor interfaces of 4.4 mm and 47% agreement. When bone was invaded with tumor, the SD of the distances increased to 5.1 mm with 9% agreement. For the other regions, the SD ranged from 6.5 to 5.6 mm.

**Table 3:** Observer variation for the various CTV elective to normal tissue interfaces and the two delineation phases.

Anatomical regions	Phase 1		Phase 2	
	
	SD (mm)	Agreement (%)	SD (mm)	Agreement (%)
All regions	5.9	17	4.9 (p = 0.01)	59 (p = 0.004)

Dorsal - Bone	4.4	47	4.2 (n.s.)	75 (n.s.)

Dorsal - Invas.	5.1	9	4.5 (p = 0.02)	43 (p = 0.01)

Pterygoid	5.6	27	4.4 (p = 0.03)	58 (p = 0.02)

Parapharyngeal	5.7	15	4.9 (p = 0.04)	53 (p = 0.01)

Sphenoid	6.1	9	5.7 (p = 0.03)	51 (p = 0.04)

Nasoph. - Lat.	5.7	11	4.7 (n.s.)	66 (p = 0.02)

Nasoph. - Ant.	6.5	10	5.1 (p = 0.02)	70 (p = 0.01)

Caudal side	7.9	7	5.6 (p = 0.03)	47 (p = 0.005)

Overall observer variation in delineation of the CTV + 1 cm, including the Nasopharynx, quadratic mean of the SD of the distance of the delineation to the median surface as measured for each region. Agreement depicts the percentage of the surface for each region on the median surface where the observers intended to delineate the same anatomical entity. Standard deviations are quadratic averages of patients specific standard deviations.

The delineations were split into two groups: those delineations where the side windows were used and those where the windows were not used by the observer. The first group had a smaller overall SD in delineation compared to the second group. The variation difference was primarily noted at the caudal and superior side of the delineations; i.e., perpendicular to the CT scan axis but in-plane for the sagittal reconstruction.

The Phase 2 delineations demonstrated a marked different SD compared to the first phase. The mean number of corrections/cm^2 ^was 10.1. The mean standard deviation of the distance between the median surface and the delineations was reduced from 5.9 to 4.9 mm, with a percentage of surface where at least 8/10 observers intended to delineate the same anatomical entity increasing from 17 to 59% (Table 3). The caudal border of the delineation was improved but there was still considerable in variation, with 5.6 mm and an agreement of 47% of the surface. The mean volume decreased from 103 to 91 cm^3 ^with a root mean square SD of 33 and 21 respectively.

## Discussion

The delineation uncertainties in this article are larger than reported uncertainties for setup error in the head and neck region. Since an error in delineation affects the whole treatment and not just one fraction it is clear that delineation is a large geometric uncertainty in radiation treatment for nasopharynx cancer [2,3,28]. This study concerns observer variation in 3D as a baseline, and aimed to reduce the target delineation variation. The results with improved consensus guidelines and matched MRI available show that the effort was successful. The mean SD of the distances decreased both for the CTV and for the CTV elective delineations. No ground truth of tumor extent was available for the patients in this study, thus no comparison tho this ground truth could be made. Still observer variation should be minimized as it has a large impact on tumor control and side effects. Furthermore, reproducible target delineations make evaluation of efficacy and side effects more precise.

The reasons for the difference between phase one and two are as follows. First, looking at the analysis of the first phase CTV delineation (baseline delineation), the largest variation was noted in the caudal direction (i.e., perpendicular to the transverse plane of the CT scan) (Table 2, 3). This was largest in those delineations where the observers did not use the (sagittal/coronal view) side windows. This is applicable to other tumor areas as well. A similar finding has been noted in delineation of lung tumors where the observer variation between observers utilizing the side windows was smaller than between the observers who did not use the side windows [13,15]. Therefore, in the second phase, the use of the side windows was enforced, by forcing the observer first to pin-point a plane in the main window where the side sagittal and/or coronal window was to be reconstructed, thus ensuring that the side window was used.

**Figure 3:**
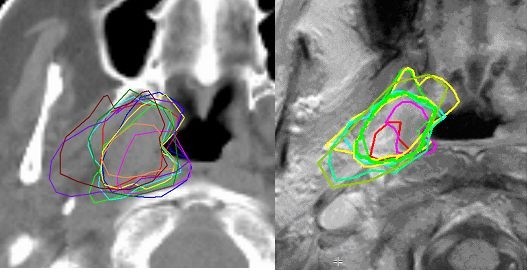
**Ten CTV delineations on one patient for Phase 1 (Left) and Phase 2 (Right)**. Each contour was delineated by a different observer.

A second source of error was found at the soft tissue boundaries of the tumor. In part, this was due to lack of soft tissue contrast in CT based delineation. In addition, as a result of the post-phase I meeting where the delineations were discussed, the issue arose regarding what to do with invaded Pterygoid muscles. Even with an agreement of the Gross Tumor Volume extension, observers disagreed upon the extent of the CTV margin into the muscle. When invaded, some considered the entire muscle or bony structure a target, while others considered a small margin around the visible tumor sufficient (Fig. 3). Apparently, the delineation instructions were unclear. As a result of this, the instructions were adapted to require inclusion of the anatomical structure if invaded by tumor, resulting in a smaller variation and an almost doubling of the agreement surface percentage. This had a large impact on the agreement between the observers.

Furthermore, in order to make more soft-tissue contrast available, without the need to view a separate MRI, a co-registered MRI was made available. Several earlier studies on nasopharynx and other head and neck regions demonstrated the superiority of MRI delineation in this respect [11,14,25,26]. To make delineation on two modalities easier, double window delineation was introduced into the second phase delineation software. With this feature, delineation in the main window (CT or MRI at the preference of the observer) was directly linked to delineation in the same plane on the other modality, allowing real time double modality delineation [13,15].

**Figure 4:**
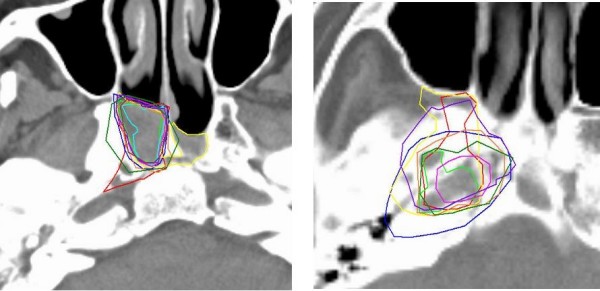
**CTV delineations on a patients with (right) and without (left) invasion of the clivus**. The delineation variation is largest when invasion was demonstrated.

The delineation of the CTV elective (CTV+ 1 cm including the Nasopharynx) showed, in part, similar results although with larger variation (Table 3). At first glance, it was unclear why this was the case. Our first expectations were otherwise, based upon earlier studies in the head and neck, where delineation of a known anatomical entity resulted in lower observer variation [28]. Analysis of the results showed a lower surface percentage of agreement, indicating that the observers *intentionally *delineated a different anatomical entity. This was the case for all interfaces. Clearly, there was a difference in interpretation of the extent of the nasopharynx. In theory, this should not have been the case, since the delineation instructions were derived from the TNM definition of the nasopharynx [29]. To rule out errors, even in the first phase of the study, the definitions were listed in the delineation instructions. Apparently, this was not sufficient. Furthermore, there was a striking difference between the observer variation towards the bone (Clivus) when it was invaded versus non-invaded parts (Fig. 4). This was demonstrated earlier by Chung et al who concluded that invasion of the clivus was best seen with the aid of MRI [26]. As a result of these findings, two measures were taken for the second delineation phase:

1 A CT-MRI atlas of the nasopharynx, with the TNM definition of the nasopharynx delineated, was available on-line for the observer. The atlas was generated from the TNM atlas and available to the observer in multiple planes.

2 The instructions for delineation of invaded bone was adapted (i.e., when the Clivus was invaded, the whole clivus was to be regarded as part of the CTV elective).

3 Forced use of sagittal windows, the observers were first to pin-point a plane in the main window where the side sagittal and/or coronal window was to be reconstructed before that no delineation could be submitted.

The sum of these measures resulted in a considerable reduction in the variation in tumor and CTV delineation. Being able to replay the delineations brought great insight into the causes of the delineation variation. One source of delineation variation (i.e., lack of soft-tissue contrast) needs an entirely different approach than do others (i.e., definition of the nasopharynx, use of sagittal windows, etc.). With clearer delineation instructions, together with the forced use of sagittal reconstructions and simultaneous delineation on CT and MRI, target delineation variation in the nasopharynx can be reduced. The largest impact on agreement was obtained by improved definitions of the CTV and CTV elective, rather than use of multimodality imaging as is most clearly demonstrated by the increase of agreement surface at the CTV elective.

## Conclusions

Observer variation of target delineation in the nasopharynx is considerable but can be reduced with the use of dedicated delineation protocols, forced use of sagittal/coronal reconstructions, and double window delineation on CT and MRI. In the current study, instructing the observers to designate the invaded structure as a target reduced an important source of variation.

## Conflict of interests

The authors declare that they have no competing interests.

## Authors' contributions

CR: primary investigator, observer, RS: co-investigator, 3D analysis, IF: co-investigator, 3D analysis, JD: design and implementation of big brother software enabling analysis of the data, PN: observer, design of the study, FPam: Radiologist, interpretation of anatomical location of variation, design of the delineation atlas, AE: observer, JK: observer, FPau: observer, MvH: supervisor, all authors read and approved the final manuscript.
